# Central corneal thickness in a Jordanian population and its association with different types of Glaucoma: cross-sectional study

**DOI:** 10.1186/s12886-018-0944-6

**Published:** 2018-10-29

**Authors:** Sana’ Muhsen, Feras Alkhalaileh, Mohammad Hamdan, Saif Aldeen AlRyalat

**Affiliations:** 1 Ophthalmology, Glaucoma and Anterior Segment Surgeon, University of Jordan Hospital, The University of Jordan, Amman, Jordan; 2 School of Medicine, The University of Jordan, Amman, 11942 Jordan; 3 Department of Ophthalmology, University of Jordan Hospital, The University of Jordan, Amman, 11942 Jordan

**Keywords:** Glaucoma, Central corneal thickness, Ethnicity

## Abstract

**Background:**

Central corneal thickness (CCT) has long been implicated to affect glaucoma predisposition. Several reports have identified that thinner CCT is a risk factor for open-angle glaucoma, and that CCT can be very variable between different ethnic groups. In this study, we aim to identify the relation between CCT and different glaucoma parameters in different types of glaucoma in an Arabian ethnicity.

**Methods:**

We classified our participants into four main groups: primary open-angle glaucoma (POAG), primary angle-closure glaucoma (PACG), pseudoexfoliative glaucoma (PXFG), and a control group. We obtained demographics, intraocular pressure (IOP), cup to disc ratio (CDR), visual field mean deviation (MD) and pattern standard deviation (PSD), CCT, and retinal nerve fiber layer (RNFL) thickness for each participant.

**Results:**

We included A total of 119 eyes with glaucoma, including POAG (54 eyes), PXFG (31 eyes) and PACG (34 eyes), we also included 57 control eyes. We found that PACG eyes have the thinnest CCT. Mean measurements of CCT for our groups were: 538.31 μm (SD = 36.30) in eyes with POAG, 544.45 μm (SD = 28.57) in eyes with PXFG, 506.91 μm (SD = 34.55) in eyes with PACG and 549.63 μm (SD = 42.9) in the control group. We found that CCT is significantly correlated with CDR (*p* = 0.012, *r* = − 0.231), MD (*p* < 0.001, *r* = 0.327),and RNFL thickness (*p* = .007, *r* = .283).

**Conclusion:**

In Arabian ethnicity, PACG patients have the thinnest CCT compared to other types of glaucoma, namely POAG and PXFG. We demonstrated that glaucomatous eyes with thinner corneas will probably have more advanced glaucomatous optic neuropathy. Our results emphasize the importance of taking ethnicity into account upon glaucoma management.

## Background

Glaucoma is the second leading cause of blindness in the world, after cataracts, and is the leading cause of blindness among African-Americans [1]. It is generally classified into open-angle and closed-angle glaucoma, and both can be either primary or secondary. It is estimated that the number of people with primary open-angle glaucoma (POAG) worldwide will reach 58.6 million by 2020, while 21 million will be affected by primary angle-closure glaucoma (PACG) [1]. Of those, 5.9 and 5.3 million will be blind from irreversible optic nerve damage associated with POAG and PACG, respectively [1]. This means that early detection and identification of risk factors are key elements in controlling the disease and preventing its progression. Secondary open-angle glaucoma is another entity with diverse types, but Pseudoexfoliation glaucoma (PXFG) is currently the leading cause of secondary open-angle glaucoma with a prevalence reaching 40% in patients over the age of 80 [2], and is highly dependent on race and ethnicity [3].

Evidence in the recent literature has shown the importance of central corneal thickness (CCT) in relation to several ocular conditions. Most notably, thinner CCT has been identified as a risk factor for open-angle glaucoma [4]. Goldmann and Schmidt discussed the association between the corneal thickness and the intra-ocular pressure (IOP) in their publication in 1957 [5]. Fourteen years later, Hansen and Ehlers demonstrated the presence of a positive linear correlation between CCT and IOP [6]. More recently, a meta-analysis of worldwide CCT literature proposed a correction factor of 2.5 mmHg for each 50 μm change in CCT [7].

CCT can be very variable between different ethnic groups, as shown in studies comparing mean CCT in both normal and glaucomatous eyes between Caucasian, Hispanic, African American, and Asians, where the thinnest corneas being more prevalent among African American ethnicity [8, 9]. Other studies also showed significant variation in CCT between ethnic sub-groups, as in a study that included different Asian populations; Chinese, Japanese, Korean, Filipino, Pacific Islander, and South Asian, and showed that the thinnest corneas are found in South Asian populations [10]. Moreover, several studies have shown that CCT varies among individuals with different types of glaucoma (POAG, PACG, and PXFG) and normal eyes, as in Bechmann et al. study that showed thinner corneas in patients with PXFG and POAG compared with patients with PACG and normal eyes [11]. In Jordanian population; an Arabian, Middle-eastern ethnicity, previous studies showed that glaucoma is one of the leading causes of blindness [12], and at the same time, the level of knowledge about glaucoma among Jordanians is poor (around 75% have either no or low level of knowledge) compared to other parts of the world [13], leading to delayed diagnosis of this silent disease. Ophthalmologists in Jordan usually depend on studies that were done on other ethnicities upon treating their glaucoma patients, due to lack of studies on the characteristics of glaucoma among Jordanians. Given the importance of early detection of risk factors of glaucoma, and as corneal thickness is one of the main risk factors for glaucoma and is the one associated with ethnicity, we aim to assess the corneal thickness in different types of glaucoma and in normal controls in a Jordanian population. We further aim to explore the relationship between CCT and the severity of glaucomatous optic neuropathy in different types of glaucoma.

## Methods

### Participants

This is a cross-sectional observational study that was conducted on subjects visiting the glaucoma clinics in 2 centers in Jordan between November 2015 and May 2017. We obtained ethical approval from the Institutional Review Board (IRB) and all patients were consented and approved to participate in this study.

This study was conducted in accordance with the Declaration of Helsinki latest update (2013). We included both patients with glaucoma and age-gender matching controls. Glaucoma patients were classified into three types of glaucoma: POAG, PACG, and PXFG. A diagnosis of POAG or PACG was made based on IOP, gonioscopy and both characteristic visual field (VF) defects (nasal step, arcuate scotoma, and paracentral defect), and optic nerve changes (enlarged cup to disc ratio (CDR), localized notch, and disc hemorrhage) in at least 1 eye as well as Ocular Coherence tomography (OCT) showing thinning of Retinal Nerve Fiber Layer (RNFL). For PXFG, pupils were dilated and exfoliation was checked and recorded as present or absent. PXFG was defined as an open-angle glaucoma with concomitant exfoliation material observed at pupillary border or anterior lens capsule with dilated pupil that is associated with glaucomatous optic neuropathy, as defined earlier.

To be included in this study the patients had to have all of the following inclusion criteria:

- Adult patients (age more than 18 years).
- A diagnosis of POAG, PACG or PXFG in at least 1 eye.
- A reliable VF examination taken within 3 months of the pachymetry. Reading had to be available for both eyes.

If the subject had any of the following exclusion criteria he/she was exempted from the study:

- Glaucoma types other than the aforementioned three types.
- Corneal pathology or surgery that might influence pachymetry.
- Underwent a cataract surgery.
- Patients with systemic diseases that might result in VF changes.
- Keratoconus.
- Contact lens use.
- Corneal dystrophy.

For controls, we included them from general ophthalmology clinics with matching gender and age (±2 years), and with a corneal topography of acceptable quality. We adopted the following exclusion criteria:

- Being a first or a second degree relative of any of our included patients.
- Keratoconus or other corneal disease
- History of Glaucoma or clinical signs of glaucoma
- History of corneal or intraocular surgery or trauma

We ran a power analysis to calculate the minimum sample size required for our study, the sample size was calculated based on the following assumptions: Effect size based on previous studies (see discussion) = 0.59; power = 80%; and two-sided alpha level = 0.05. The required total sample size was 90.

### Parameters measured

Diagnosis of glaucoma and classification was confirmed by the attending ophthalmologist based on IOP measurements, gonioscopy, optic nerve changes, visual field defects and OCT RNFL thinning as stated before. We also asked included patients about their demographic data including age, gender, and ethnicity, as well as their previous ocular and medical history.

We measured the IOP using a Goldmann applanation tonometer, where our consultant ophthalmologist performed three measurements for each participant, and the average value was calculated. IOP readings included in the analysis were of patients treated with one or more antiglaucoma eye drops. Gonioscopy was done using a 4-mirror Sussman lens to classify angles into open or closed and to illicit signs of PXFG. A detailed anterior segment examination by slit lamp was performed to rule out corneal pathology that would exclude the patients from the study and also to help detect pseudoexfoliation. Optic nerve assessment including cup-to-disc ratio (CDR) using indirect biomicroscopy and a super field lens were performed by the consultant ophthalmologist.

We measured CCT via Oculus Pentacam HR for both glaucoma patients and controls. The Pentacam HR is a high-resolution rotating Scheimpflug camera system for anterior segment analysis. It provides crisp images of cornea, iris and lens. An ophthalmic technician performed the imaging during daytime (from 9:00 am to 4:00 pm). We then analyzed the Pentacam printouts to rule out any corneal pathologies, such as keratoconus, that would exclude patients from our study and to determine the central corneal thickness at the apex.

Other parameters were assessed for glaucoma patients only. Visual Field assessment was performed using Oculus Centerfield analyzer with a screening 24-2 Threshold strategy. RNFL thickness measurement was performed using Optovue RTVue Optical Coherence Tomography OCT, which generates high-resolution, cross-sectional (3D) images of the retina, optic disc and anterior segment.

### Statistical analysis

We used SPSS 21.0 (Chicago, USA) in our statistical analysis. We first described our sample population and eyes included via descriptive statistics including numbers (percentages) and mean (+\- standard deviation SD).

We used logistic regression with generalized estimating equation to analyze effect of gender on type of glaucoma developed and eyes included. We used Independent sample t-test to study differences in general parameters and both the gender and the eye involved. To account for the within-subject effect, we used one-way repeated measure univariate analysis to study the relation between type of glaucoma and general parameters, followed by post-hoc analysis using Tukey analysis. We used Pearson’s correlation to study the correlation between age and the other parameters. We also performed Spearman’s test, after controlling for gender, to confirm association results. We finally adopted a model building strategy for a regression analysis to find predictors of visual field mean deviation (MD), where we corrected for age and gender. We inspected our data visually using boxplots and histograms, and we used Levene’s test to check for homogeneity of variance. We also used Mauchly’s test for sphericity to apply univariate analysis. We used a threshold of 0.05 for *p* value to indicate statistical significance.

## Results

We included a total of 68 Jordanian patients in this study, with a mean age of 65.9 years (SD = 8.9). There were 42 (62%) men and 26 (38%) women. From the sample included, 119 eyes met our inclusion criteria and were included in our study, they were 61 (51.3%) right eyes and 58 (48.7%) left eyes (51 bilateral and 17 unilateral eyes). Eyes with glaucoma were categorized according to type of glaucoma into: POAG (54 eyes), PXFG (31 eyes) and PACG (34 eyes), as shown in (Table 1). We also included 29 control participants with a mean age of 55 years. They were 14 men and 15 women and a total of 57 eyes.

**Table 1:** General descriptive for the main three types of glaucoma and control group

	Type of Glaucoma			Control
	Primary Open Angle Glaucoma	Closed Angle Glaucoma	Pseudoexfoliation Glaucoma	
Gender				
Male Frequency (%)	41 (75.9%)	14 (41.2%)	19 (61.3%)	27 (47.4%)
Female Frequency (%)	13 (24.1%)	20 (58.8%)	12 (38.7%)	30 (52.6%)
Age				
Mean (±SD)	64.26 (±9.18)	66.85 (±9.98)	67.8 (±6.93)	54.85 (±10.9)
Eye				
Right Frequency (%)	27 (50%)	18 (52.9%)	16 (51.6%)	28 (49.1%)
Left Frequency (%)	27 (50%)	16 (47.1%)	15 (48.4%)	29 (50.9%)

Upon analyzing the correlation between gender discrepancy and type of glaucoma developed, we found a significant difference (*p* < 0.001) as men were more likely to develop POAG (55.4%) compared to 28.9% for women. However women were more likely to develop PACG (44.4%) compared to only 18.9% for men. No significant difference was found between gender discrepancy and the development of PXFG. We found no statistically significant difference between both genders regarding other parameters. Mean values of different glaucoma-related parameters for both genders are detailed in (Table 2).

**Table 2:** Gender differences of the mean glaucoma parameters measured in this study among glaucoma patients

	Gender	Mean	Std. Deviation	Std. Error Mean
Intra-ocular Pressure	Male	15.1739	4.49347	0.66253
	Female	15.6724	5.12744	0.95214
Cup to Disc Ratio	Male	0.6234	0.27892	0.03242
	Female	0.5587	0.24368	0.03633
Mean Deviation	Male	−5.5229	5.97786	0.71965
	Female	−6.6430	5.83435	0.88973
Pattern Standard Deviation	Male	3.9250	1.72405	0.21222
	Female	4.2298	1.51917	0.23167
Retinal Nerve Fiber Layer Thickness	Male	77.5424	21.27373	2.47302
	Female	78.3881	19.10243	2.91309
Central Corneal Thickness (CCT)	Male	534.5270	34.14763	3.96958
	Female	525.0444	41.09797	6.12652

Regarding central corneal thickness (CCT), our results showed the following mean measurements: 538.31 μm (SD = 36.30) in eyes with POAG, 544.45 μm (SD = 28.57) in eyes with PXFG, 506.91 μm (SD = 34.55) in eyes with PACG, and 549.63 μm (SD = 42.9) in the control group. First, we studied the difference between CCT of each glaucoma type and the control group, we found that subjects with PACG had significantly thinner corneas than the control group (*p* = 0.021). No statistically significant difference was found between CCT of control group and any of POAG or PXFG groups (*p* = .702). Upon comparing mean CCT between glaucoma groups, we found a significant difference between the groups in general (*p* < 0.001), so we performed a post-hoc analysis to find the group differences as shown in (Table 3).

**Table 3:** Relation between type of glaucoma and different parameters

Parameter	Primary Open Angle Glaucoma	Closed Angle Glaucoma	Pseudoexfoliative Glaucoma	Between subject effect *p*-value	Mean difference (95% CI)	Post-hoc *p*-value
Intraocular pressure (mmHg)	15.2 (4.3)	13.6 (3.2)^*^	17.3 (5.8)^*^	0.049	* 3.7 (0.1–7.2)	*0.04
Cup to disc ratio	0.61 (0.25)	0.72 (0.18)^*^	0.44 (0.29)^*^	0.006	* 0.3 (0.8–0.47)	*0.004
Retinal nerve fiber layer thickness (μm)	80.5 (20.8)	70.8 (18.2)	82.2 (20.9)	0.084	–	–
Central corneal thickness (μm)	538.3 (36.3)^*^	506.9 (34.6)*^#^	544.5 (28.6)^#^	< 0.001	* 46.2 (21. 9-70.6) # 36.6 (10. 5-62.7)	* < 0.001 # 0.004
Visual field mean deviation (dB)	−4.9 (5.6)^*^	−9.0 (6.8)^*#^	−4.7 (4.4)^#^	< 0.001	7.3 (3. 5-11.1) #7.5 (3. 5-11.6)	* < 0.001 # < 0.001
Visual field Pattern standard deviation	4.0 (1.7)	4.1 (1.8)	4.1 (1.5)	0.351	–	–

Mean reading for each parameter and its standard deviation are presented. Significant differences between any two readings as analyzed by post-hoc analysis were marked by either (*) or (#)

Upon studying the relation between CCT and parameters of optic nerve damage (CDR, MD, and RNFL) in patients with glaucoma, we found that CCT is negatively and significantly correlated with CDR (*p* = 0.012, *r* = − 0.231), so that the thinner the cornea, the higher the CDR. We also found a significantly positive correlation between CCT and MD (*p* < 0.001, *r* = 0.327), so that the thinner the cornea the lower the MD (more advanced visual field loss). Finally, we found a positive correlation between CCT and RNFL (*p* = .007, *r* = .283) meaning that the thinner the cornea, the thinner the RNFL. No significant correlation was found between CCT and either IOP or pattern standard deviation (PSD).

We studied factors predicting visual field mean deviation (MD), and found that it is significantly associated with both CDR (*p* < 0.001) and RNFL (*p* = 0.003). Upon analyzing the relation between type of glaucoma and our measured parameters, we found several significant relations; our results are summarized in (Table 2). No statistical significance was found between either the age or laterality of the involved eye (left or right) on one hand and type of glaucoma or other measured parameters on the other hand.

## Discussion

**Fig. 1:**
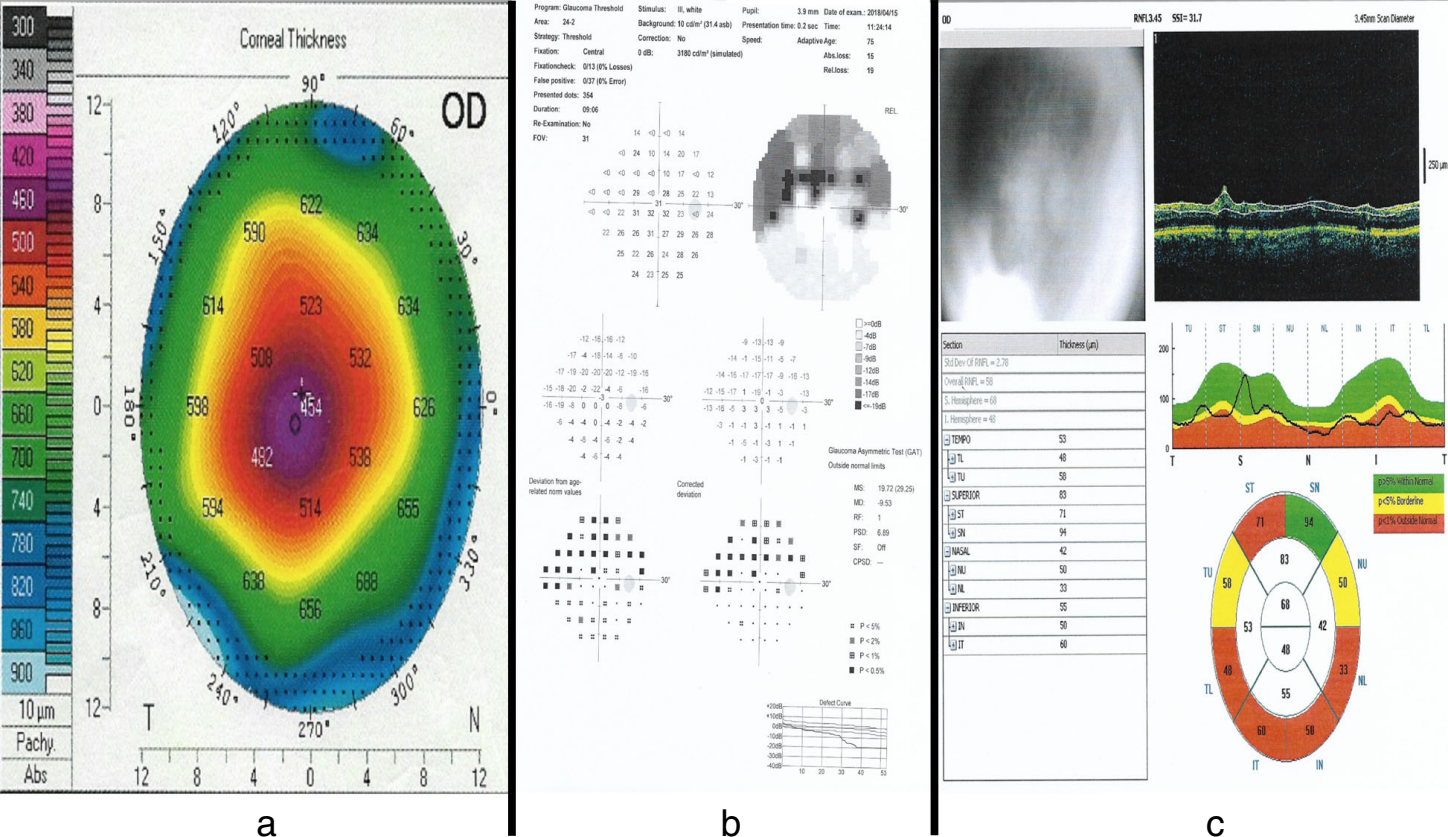
Imaging in a patient with advanced chronic angle closure glaucoma. **a** Pentacam printout of right eye corneal thickness map showing a central corneal thickness (CCT)of 454 μm, which is significantly thinner than average CCT of 530–550 μm. **b** Threshold 24–2 Visual Field test of the right eye demonstrating a superior arcuate scotoma splitting fixation. **c** Ocular coherence tomography of the retinal nerve fiber layer thickness (OCT RNFL) of the right eye showing significant thinning (Overall RNFL = 58).

This study was done on a Jordanian population, a poorly studied population regarding glaucoma and its risk factors. The mean central corneal thicknesses (CCT) of normal adults and patients with 3 different types of glaucoma was measured by corneal topography. We also established that CCT in patients with PACG is significantly thinner than in other glaucoma types and in the control group. As visual field defect is an important outcome in glaucoma, we found that both cup to disc ratio (CDR) and retinal nerve fiber layer (RNFL) thickness, as measured by Optical Coherence Tomography, are the main factors associated with lower mean deviations on visual field testing. Gender discrepancy in glaucoma type preferences found to be in concordance with some previous reports where men are more likely to develop POAG [14], and women are at higher risk for PACG, likely due to anatomical predisposition [15]. (Fig. 1) shows images for a patient with advanced chronic angle closure glaucoma and with significantly thin central corneal thickness, where the visual field test demonstrating a superior arcuate scotoma splitting fixation and ocular coherence tomography of the retinal nerve fiber layer thickness (OCT RNFL) shows significant thinning.

Previous studies, that were done on western populations, aimed to find the relation between CCT and other parameters, including visual field defects, have mainly included POAG eyes, and they found significantly more advanced visual field defects for lower CCT compared to thicker ones [16, 17]. Here, we studied different types of glaucoma patients and control participants from a Jordanian population, and we found similar correlations where patients with lower CCT had significantly more advanced visual field defects, and thinner RNFL. We also found that patients with PACG had the lowest CCT compared to POAG, PXFG, and controls. An Iranian study, that included both POAG and PACG patients, found that POAG patients have lower CCT compared to their PACG counterparts [18]. Regarding PXFG, a Caucasian study found a significantly lower CCT in PXFG patients compared to POAG patients [19], this emphasizes the profound difference in CCT in different ethnic groups and the importance of CCT as a risk factor for progression of glaucoma.

The strong relation between CCT and ethnicity is well-studied. A large study that was done on a Chinese population to study the relation between CCT and around 20 variables found that ethnicity is one of the important determinants of CCT [20]. A worldwide meta-analysis found that the average CCT for normal eyes derived from various racial groups, regardless of the device used, was 544 μm [21], a value that is thinner than that for the Jordanian population we studied (550 μm). Comparing CCT values of our Jordanian population with Caucasian ethnicity, the most represented ethnicity in ethnicity-comparing studies [22], Caucasian ethnicity mean CCT was similar to Jordanian’s (550 ± 30.5 μm) [23]. On the other hand, African ethnicity has a lower CCT than that for Jordanians (514.77 ± 31.86) [23]. It should be noted that the technique used to measure CCT can affect the results [24], and although the results from commonly used devices mostly correlate, the direct comparison between them might not be accurate [22], so that we will only include studies that used similar techniques for direct value comparison purposes. Regarding CCT difference between different glaucoma types, only PACG eyes have thinner corneas compared to POAG, PXFG, and control eyes, This comes in accordance with what a recent study suggested, where no difference in corneal thickness between POAG, PXFG, and control eyes was found [25].

Other reports generally have results that are comparable to ours, except for a study by Tolesa and Gessesse that was done on Ethiopian patients [26], and found thicker CCT in patients with POAG (520 ± 38.95) compared to PXFG patients (507 ± 35).

Several previous studies have reported the association between larger CDR and glaucomatous progression [27]. Here, we found that patients with larger CDR have worse mean deviation (MD) compared to those with smaller ratios. In Ocular Hypertension Treatment Study [28], they found that cup to disc ratio is also a predictor of the development of POAG in normal subjects. A recent study found that for every 0. 1-unit increase in the CDR, there is an increase in the decay rate of visual field by 23% [29]. We also found that the largest CDR is found in PACG patients, followed by POAG, and finally PXFG.

Similarly, we found that RNFL thickness measured by Optical Coherence Tomography (OCT) is also a good indicator for the severity of visual field loss represented by the mean deviation (MD). In a 12-year study, the risk estimate for moderate and severe visual field defects found to suggest a seven to eight times greater likelihood for future field loss for eyes with initial atrophy [30]. Where previous reports found an association between IOP and MD [30], we think that this effect is indirect through affecting the CDR, as the relation between IOP and MD did not reach significance when we corrected for CDR, probably because these were treated IOP readings.

Several limitations of this study exist. We advice future studies to include only one eye for each included subject (independence of observation) to overcome correlated observation issue as explained in a previous study, although it can be corrected using certain statistical tests [31]. Moreover, a longitudinal approach to follow Middle Eastern patients would provide a better insight on the outcome of different CCT. Although this is a multicenter study, future studies from other Arabian countries should consider including larger sample size to confirm the generalizability of our results.

## Conclusion

We found that PACG patients have the thinnest CCT compared to other types of glaucoma, namely POAG and PXFG. We also demonstrated that glaucomatous eyes with thinner corneas will probably have more advanced glaucomatous optic neuropathy. This emphasizes the importance of making CCT measurement a part of standard care in all glaucoma patients since it can predict patients with worse prognosis, and therefore can indicate the need for more aggressive management and earlier diagnosis. Finally, we showed that cup to disc ratio, retinal nerve fiber layer thickness and mean deviation on visual field testing usually are in concordance regarding the severity of glaucoma.

## Abbreviations

CCT: Central corneal thickness
CDR: Cup-to-disc ratio
IOP: Intraocular pressure
MD: Visual field mean deviation
OCT: Ocular coherence tomography
PACG: Primary angle closure glaucoma
POAG: Primary open angle glaucoma
PSD: Pattern standard deviation
PXFG: Pseudoexfoliative glaucoma
RNFL: Retinal nerve fiber layer
